# A Multicenter Trial of an Enhanced Serum Comprised of 13 Plant‐Based Adaptogens Targeting Skin Quality in Females Impacted by Hormonal Decline

**DOI:** 10.1111/jocd.70802

**Published:** 2026-05-12

**Authors:** Zoe D. Draelos, Amir Moradi, Amber Smathers

**Affiliations:** ^1^ Dermatology Consulting Services PLLC High Point North Carolina USA; ^2^ Moradi MD, Cosmetic Surgery Vista California USA; ^3^ Skinbetter science, Dermatological Beauty Brand of L'Oréal USA Inc. New York City New York USA

**Keywords:** adaptogens, antioxidants, hormonal fluctuations, menopause

## Abstract

**Objectives:**

To assess the effectiveness and tolerability of a serum comprised of plant‐based adaptogens (MYS‐REV) for visible improvements in skin changes caused by hormonal fluctuations associated with perimenopause and postmenopause in women.

**Study Designs:**

A 16‐week open‐label multicenter study evaluated the efficacy and tolerability of twice‐daily application of MYS‐REV in females, aged ≥ 46 years, Fitzpatrick skin type (FST) I–VI, with fine‐to‐moderate depth lines and wrinkles, mild‐to‐moderate elastosis, and crepey skin. Investigators assessed improvements in depth and number of lines/wrinkles, elastosis, and crepey skin. Additionally, investigators assessed improvements in appearance of skin dullness, skin roughness/texture, erythema, uneven pigmentation, and pore size, along with objective changes in skin hydration and transepidermal water loss (TEWL), all over 16 weeks. Tolerability and subject self‐assessment were assessed over 16 weeks.

**Results:**

At 16 weeks, twice‐daily use of MYS‐REV demonstrated mean percent improvements in elastosis/crepey skin, lines/wrinkles, skin hydration, and TEWL, and in erythema, dullness, texture/roughness, pore size, and uneven pigmentation (all *p* < 0.0001). All AEs were mild with no study discontinuations owing to any AE. Subjects reported high levels of satisfaction with MYS‐REV beginning as early as 4 weeks and continuing through Week 16.

**Conclusions:**

This study demonstrated that twice‐daily application of MYS‐REV visibly improves skin affected by menopause‐related hormonal fluctuations.

## 1 Introduction

Women experience hormonal fluctuations throughout their reproductive life, beginning with menarche and culminating with menopause at an average age of 51 years in the US [1]. The transitional period prior to menopause, or perimenopause, lasts approximately 4–8 years and is defined by diminishing levels of estrogen and progesterone. While most women focus on the resulting vasomotor symptoms (hot flashes and night sweats) and irregular menstrual bleeding patterns, these hormonal fluctuations also have a substantial impact on skin. Estrogen, and particularly 17‐β estradiol, plays an integral role in the maintenance of skin health, as it influences collagen and elastin synthesis, hydration, and overall skin vitality. However, diminishing levels of 17β estradiol loss associated with aging and the menopausal transition lead to important functional and structural changes to skin [2], including reduced collagen, elastin, and fibroblast function [3], altered ceramide production [4], decreased hydration [5], impaired barrier function, and decreased antioxidant capacity [3]. Diminishing collagen and elastin fibers in the dermal layer due to decreasing 17β estradiol levels result in clinical manifestations including fine lines/wrinkles, crepiness and sagging skin, skin dryness, skin dullness, erythema, and rough skin texture [3, 6].

Plant adaptogens are natural components or extracts found to target multiple pathways, support the body's adaptive defense communication and the body's resistance to negative stressors, and foster balance [7]. A novel serum (MYS) was developed using proprietary P.A.T.H. (Plant Adaptations Targeting Homeostasis) technology that combined 9 plant adaptogens targeting homeostasis. The serum, which is appropriate for all skin types, supports skin's natural ability to achieve balance. The initial 12‐week clinical trial involving 53 subjects with predominantly mild‐to‐moderate (81%) facial photodamage showed twice‐daily application of MYS led to visible improvements in skin quality and significant mean percent improvements from baseline in erythema, dullness, texture, pore size, and uneven pigmentation (all *p* < 0.0001), with mild and transient adverse events [8].

An enhanced serum (MYS‐REV) was developed combining P.A.T.H. and TAP technologies (topical Allyl PQQ; pyrroloquinoline quinone allyl ester) to restore, revitalize, and rebalance skin affected by hormonal fluctuations associated with menopause (Table 1). MYS‐REV uses P.A.T.H.[13] biotechnology and is formulated with 13 plant adaptogens selected for their known ability to harmonize the skin pathways impacted by these hormonal fluctuations. As mitochondrial free radical production increases with age [9, 10, 11], TAP biotechnology targets intrinsic free radicals to mitigate oxidative stress. Additional key ingredients provide barrier support (cholesterol, ceramides), hydration (glycerine, hyaluronate, niacinamide), and antioxidant benefits (alpha lipoic acid [thioctic acid], astaxanthin, ergothioneine, superoxide dismutase (SOD), and alpha‐tocopherol [vitamin E]). The combined enhanced serum specifically targets dry‐to‐very dry, perimenopausal and postmenopausal skin.

**TABLE 1:** Key biotechnology ingredients in MYS‐REV.

	Key ingredients	Benefits
P.A.T.H.[13] technology	*Panax ginseng* extract, cocoa leaf cell extract, hops extract, *Echinacea purpurea* extract, matricaria flower extract, turmeric root extract, magnolia officinalis bark extract, balsam copaiba resin, *Sophora japonica* bud extract, black currant seed oil, evening primrose oil, grape flower cell extract, and sea buckthorn	A proprietary and powerful blend of plant‐based adaptogens designed to target pathways and enhance skin's natural ability to adapt to stress and achieve balance (homeostasis) for healthier‐looking skin that glows. Helps minimize dryness, softens the appearance of fine lines and wrinkles, and promotes a more revitalized appearance.
TAP technology	Allyl PQQ (pyrroloquinoline quinone allyl ester)	Patented, potent antioxidant technology effectively neutralizes intrinsic free radicals to minimize the effects of oxidative stress.
Barrier support	Cholesterol	A key lipid that aids in reinforcing the skin's barrier, preventing moisture loss, and protecting against external aggressors.
	Ceramides EOP & NP	Help replenish moisture and reinforce the skin's barrier.
Hydration	Glycerin	Humectant with water‐binding properties that helps deliver intense hydration.
	Hydrolyzed sodium hyaluronate	Known to provide antioxidant support and moisture.
	Niacinamide	Form of vitamin B3, helps strengthen the skin's moisture barrier.
Antioxidant	Alpha lipoic acid (thioctic acid)	A potent antioxidant that helps deter against signs of photoaging, improving skin's overall quality, and appearance.
	Astaxanthin	Carotenoid from algae that contributes to the unique color of the product.
	Ergothioneine	An amino acid and powerful antioxidant that energizes the skin.
	Superoxide Dismutase	Soothes and improves the overall appearance of skin.
	Tocopherol	Alpha‐tocopherol, the most biologically active form of vitamin E, is a powerful antioxidant that neutralizes free radicals.

As part of a research program evaluating the efficacy, safety, and tolerability of MYS‐REV, we first sought to compare its effect on biomarkers known to be influenced by hormonal fluctuations. We compared MYS‐REV against a prescription topical estrogen (RX Topical Estradiol 0.015%), a leading competitor (estrogen analog), and control vehicle (0.9% saline) using a 3D in vitro skin model containing epidermal keratinocytes and dermal fibroblasts (MatTek EFT‐400). All test materials showed antioxidant effects, albeit through the expression of different sets of genes. Compared to saline control, MYS‐REV significantly increased the hydration gene aquaporin‐3 (AQP3; 209%), antioxidant/stress response genes superoxidase dismutase 2 (SOD‐2; 78%) and thioredoxin reductase 1 (TXNRD1; 358%), growth factor gene heparin‐binding epidermal growth factor (HBEFG; 821%), and the epidermal barrier genes involucrin (IVL; 131%) and occludin (OCLN; 95%); all *p* < 0.05. There was no effect from the topical estradiol on hydration but similar benefits on epidermal barrier genes, whereas the estrogen analog increased hydration but had a minimal impact on epidermal barrier genes. In summary, MYS‐REV demonstrated significant benefits on growth factor genes and those associated with hydration, epidermal barrier health, and stress response.

Herein, we initiated a clinical study to evaluate the effectiveness, safety, and tolerability of MYS‐REV on clinical manifestations of hormonal fluctuations in perimenopausal and postmenopausal females.

### 1.1 Study Objectives

Evaluate the safety, tolerability, and efficacy of an enhanced serum containing plant adaptogens (MYS‐REV) in perimenopausal and postmenopausal females.

## 2 Design and Methods

This multicenter, open‐label 16‐week trial enrolled 55 perimenopausal and postmenopausal females, ≥ 46 years of age, FST I‐VI. The study was conducted under institutional review board approval (Sterling; Atlanta, GA) in conjunction with current Good Clinical Practice (cGCP) guidelines. All subjects provided written consent to participate in the study and for the photographic release of their images for research, publication, and commercial purposes. The target recruitment goal was to enroll 1/3 perimenopausal women, 1/3 women < 5 years postmenopause, and 1/3 women > 5 years postmenopause. Eligibility criteria included having dry, normal, and sensitive skin and fine‐to‐moderate depth lines/wrinkles (Score 3–6) and mild‐to‐moderate elastosis and crepey skin (Score 3–6) on a Fitzpatrick–Goldman Classification of Wrinkling and Degree of Elastosis Scale [12] with at least mild (2) to moderate (3) signs of the following: skin dullness, skin roughness/texture (visual), erythema, uneven pigmentation, and pore size (Score of 0 = None to 5 = Very Severe). All subjects agreed to use only the sponsor‐provided skincare products, which included a gentle cleanser, moisturizer, and SPF 56. All subjects were willing to minimize UV exposure and avoid use of self‐tanners; agreed to daily use of sunscreen applied 15 min prior to being outside and reapplied every 2 h as needed, and to wear sun‐protective clothing.

Subjects with any dermatologic disorder which, in the investigator's opinion, may interfere with the accurate evaluation of the subject's facial skin, including severe lines/wrinkles, elastosis (skin laxity), erythema, roughness/texture, dullness, pore size, and uneven pigmentation, as well as the presence of prominent false lashes, were excluded from study participation. Subjects with severe rosacea or telangiectasia, moderate‐to‐severe acne, melasma, PIH, scarring, psoriasis, atopic dermatitis, or lupus erythematosus were excluded from study participation. Hormone replacement therapy (HRT) replaces depleted estrogen to more youthful levels. It has been shown to directly impact skin by facilitating increased epidermal hydration, skin elasticity, and skin thickness [13]. HRT also minimizes skin wrinkles [14], and improves the collagen content and quality as well as vascularization [15]. As such, to accurately evaluate the effects of this product on expected skin changes from hormonal fluctuations in menopause, subjects on HRT (whether oral, topical, pellets, or implants), unless to treat infertility (> 10 years prior), were excluded from study participation. Subjects with any use of isotretinoin or weight loss medications or starting a weight loss program were also excluded from study participation.

Subjects were deemed eligible for inclusion following a two‐week washout of supplements for hormonal decline; nonprescription topical retinoids, growth factors, peptides, alpha hydroxy acids (AHAs), beta hydroxy acids (BHAs), antioxidants, or vitamin C. Subjects were deemed eligible for inclusion following a four‐week washout of prescription topical products including vitamin A derivatives or hydroquinone. Subjects who had undergone microdermabrasion, microneedling, chemical peels, or like procedures; used topical prescription corticosteroids or medications for rosacea, or who had participated in other facial topical or cosmetic product studies within the prior 3 months were excluded from the study. Subjects who had used injectable neuromodulators or dermal fillers within the prior 6 months, or who had undergone any energy‐based treatments, were also excluded from study participation. Subjects were eligible for participation if they had not used any oral or transdermal hormonal contraceptives, vaginal ring, hormonal IUD, progestin implant, or injection within the prior 5 years.

After using a cleanser (AM/PM), subjects were instructed to apply the study product (MYS‐REV) twice‐daily for 16 weeks followed by a moisturizer (AM/PM) and sunscreen (AM). Investigator evaluated depth and number of lines and wrinkles, elastosis (skin laxity), and crepey skin using a Fitzpatrick–Goldman Classification of Wrinkling and Degree of Elastosis Scale [Score, 1–9] following twice‐daily use of MYS‐REV from baseline over 16 weeks. Investigator assessment of facial skin parameters including skin dullness, skin roughness/texture (visual), erythema, uneven pigmentation, and pore size from baseline over 16 weeks utilized six‐point grading scales (0 = None to 5 = Very Severe) at baseline and Weeks 4, 8, 12, and 16. Investigator assessment of safety was based on degree of erythema, edema, and dryness/flaking at baseline prior to the initial in‐clinic application, and at Weeks 4, 8, 12, and 16 using a four‐point grading scale (0 = None to 3 = Severe). Subject self‐assessed tolerability was based on sensation of burning, stinging/tingling, and itching using a four‐point grading scale (0 = None to 3 = Severe) at baseline prior to the initial in‐clinic application, and at Weeks 4, 8, 12, and 16. Changes in skin hydration (Corneometer) and transepidermal water loss (Evaporimeter) measurements were evaluated using bioinstrumentation in a subset of subjects over 16 weeks prior to and immediately following in‐clinic application at baseline, and Weeks 4, 8, 12, and 16. Subjects completed self‐assessment questionnaires at 4, 8, 12, and 16 weeks. Adverse events (AEs) were collected throughout the study period.

## 3 Results

### 3.1 Demographics

Fifty five subjects were enrolled and 53 completed the study; two discontinued. Subjects ranged from 47 to 67 years, with a mean age of 55 years. Nearly one‐third (29%) were perimenopausal; 17% were recently menopausal (< 5 years) and 54% were postmenopause ≥ 5 years. The majority of subjects (78%) were Caucasian, 13% were African American, and 5% Asian/Pacific Islander; 92% were non‐Hispanic. Similarly, most subjects (78%) were FST I–III, while 22% were FST IV–VI (Table 2).

**TABLE 2:** Demographics.

Baseline demographics (*N* = 53)		
Mean age (age range)	55 years (47–67)	
Menopausal status	Perimenopausal: 29%	Postmenopausal: 71%
Fitzpatrick skin type	FST I: 26% FST II: 43% FST III: 9%	FST IV: 7% FST V: 11% FST VI: 4%
Race	Caucasian: 78% African American: 13%	Asian or Pacific Islander: 5% Other: 4%

### 3.2 Efficacy

**FIGURE 1:**
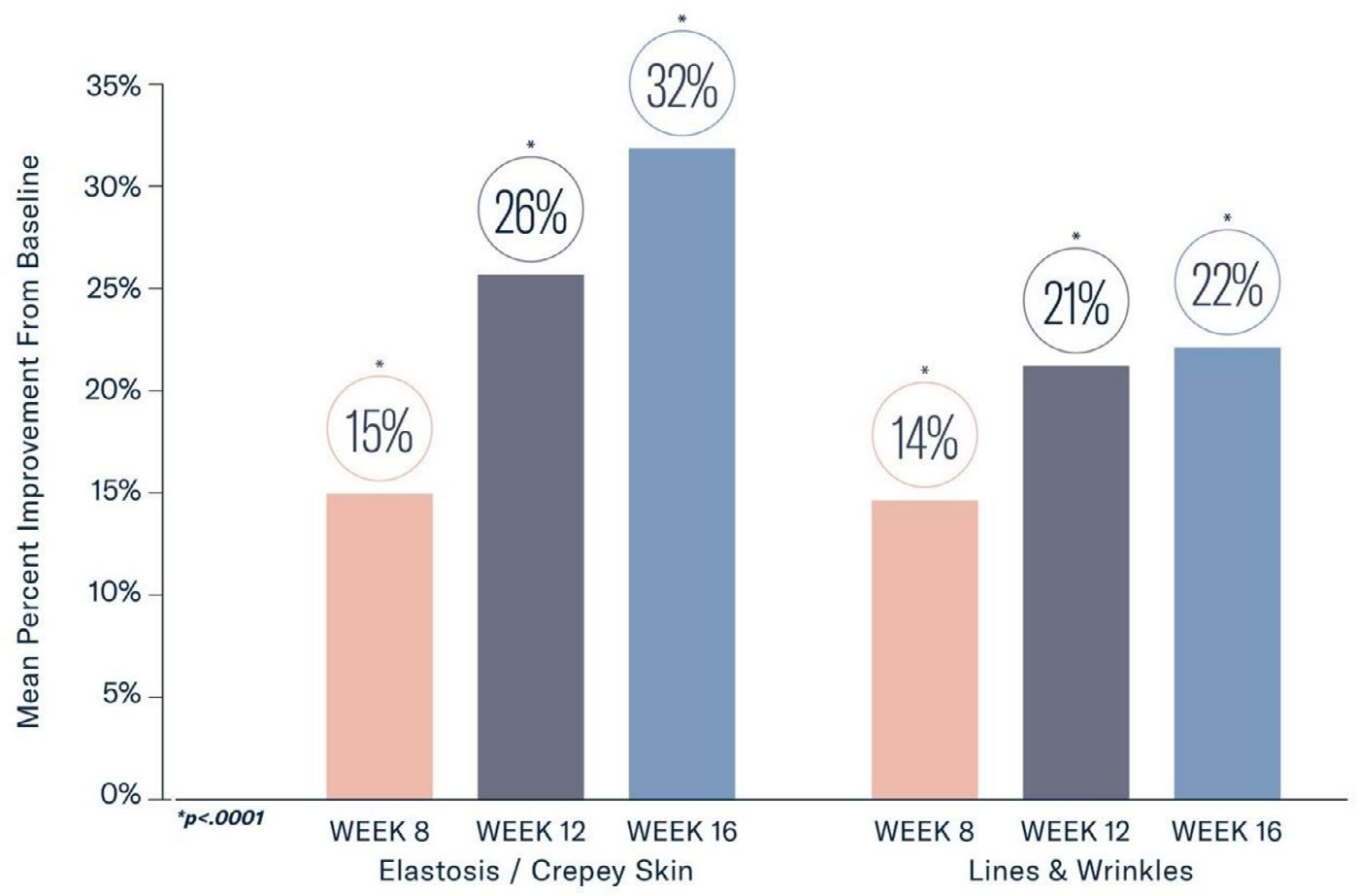
Significant mean percent improvements were demonstrated in elastosis/crepey skin and lines and wrinkles from baseline at Weeks 8, 12, and 16 (all *p* < 0.0001).

**FIGURE 2:**
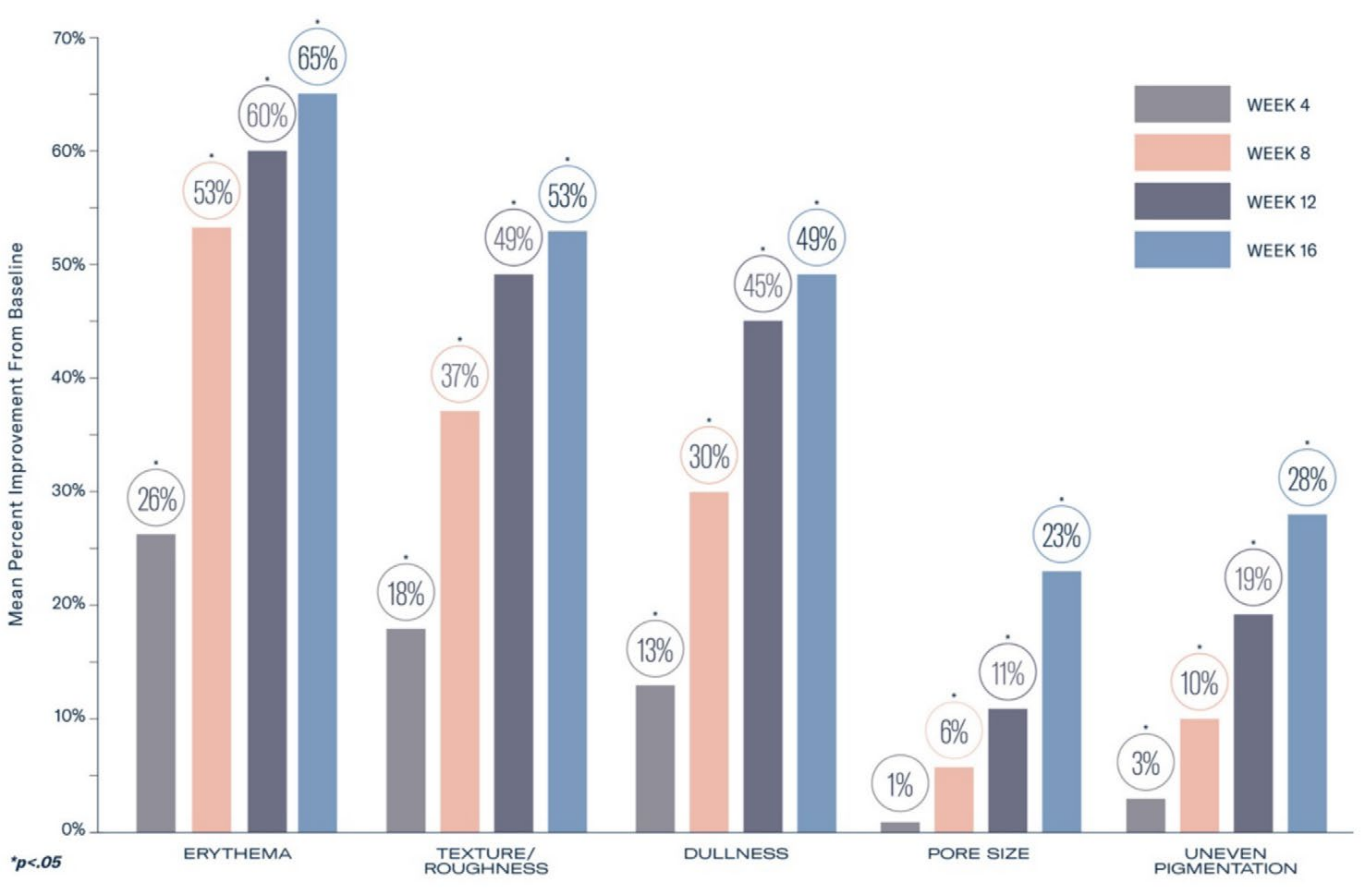
Significant mean percent visible improvements were demonstrated in all skin quality parameters evaluated including erythema, texture/roughness, dullness, pore size, and uneven pigmentation from baseline at Week 16 (*p* < 0.05).

There were nonsignificant 1% improvements in both elastosis/crepey skin and lines and wrinkles at 4 weeks. The mean percent improvement in both elastosis/crepey skin and lines and wrinkles was statistically significant as early as Week 8 (15% and 14%, respectively), with continued improvement through Week 16 (32% and 22%, respectively; all *p* < 0.0001; Figure 1). Significant mean percent improvements from baseline were observed at Week 16 in erythema (65%), dullness (49%), texture/roughness (53%), pore size (23%), and uneven pigmentation (28%; all *p* < 0.0001; Figure 2).

**FIGURE 3:**
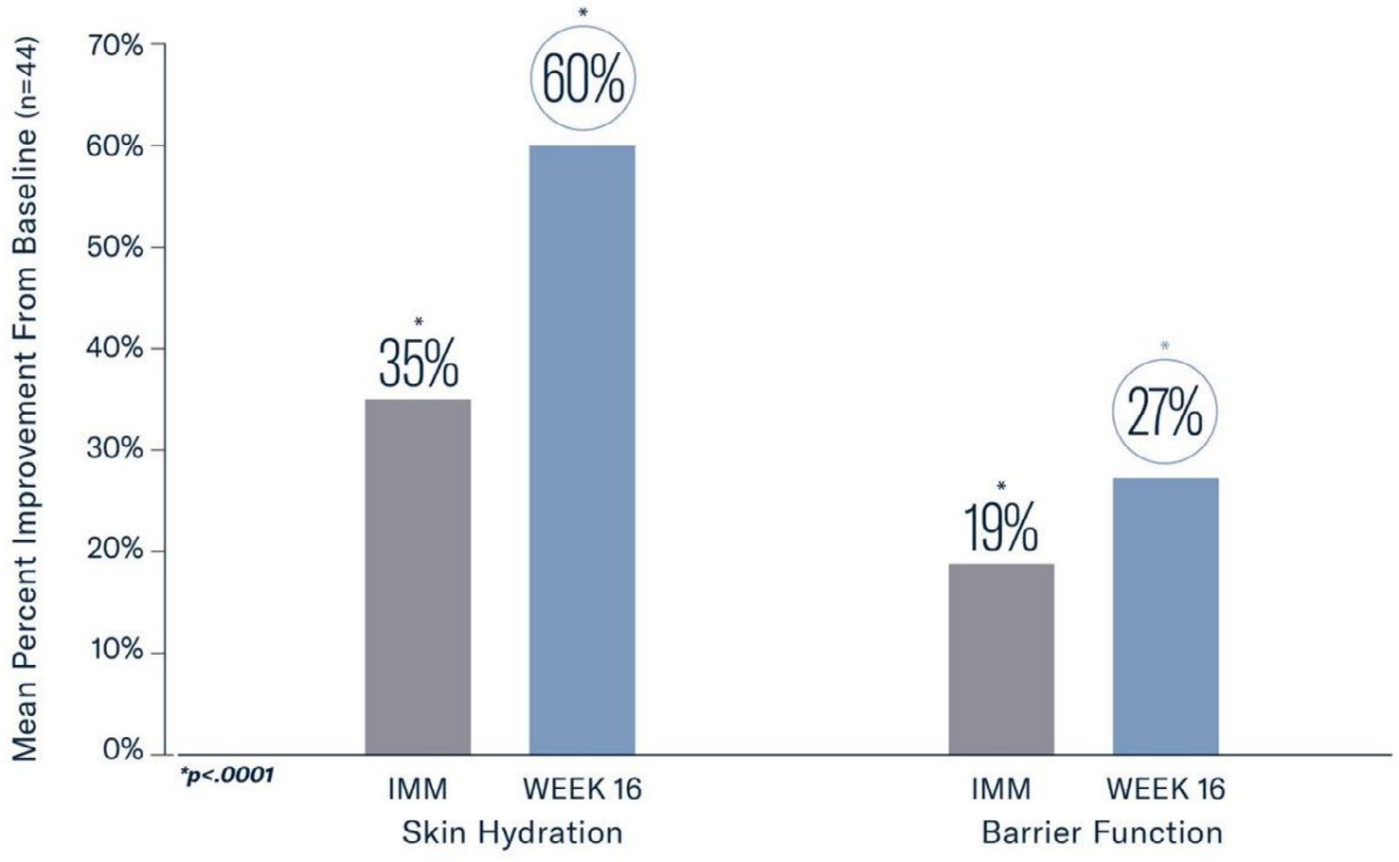
Significant improvements were demonstrated in skin hydration and barrier function at Week 16 (*p* < 0.0001).

**FIGURE 4:**
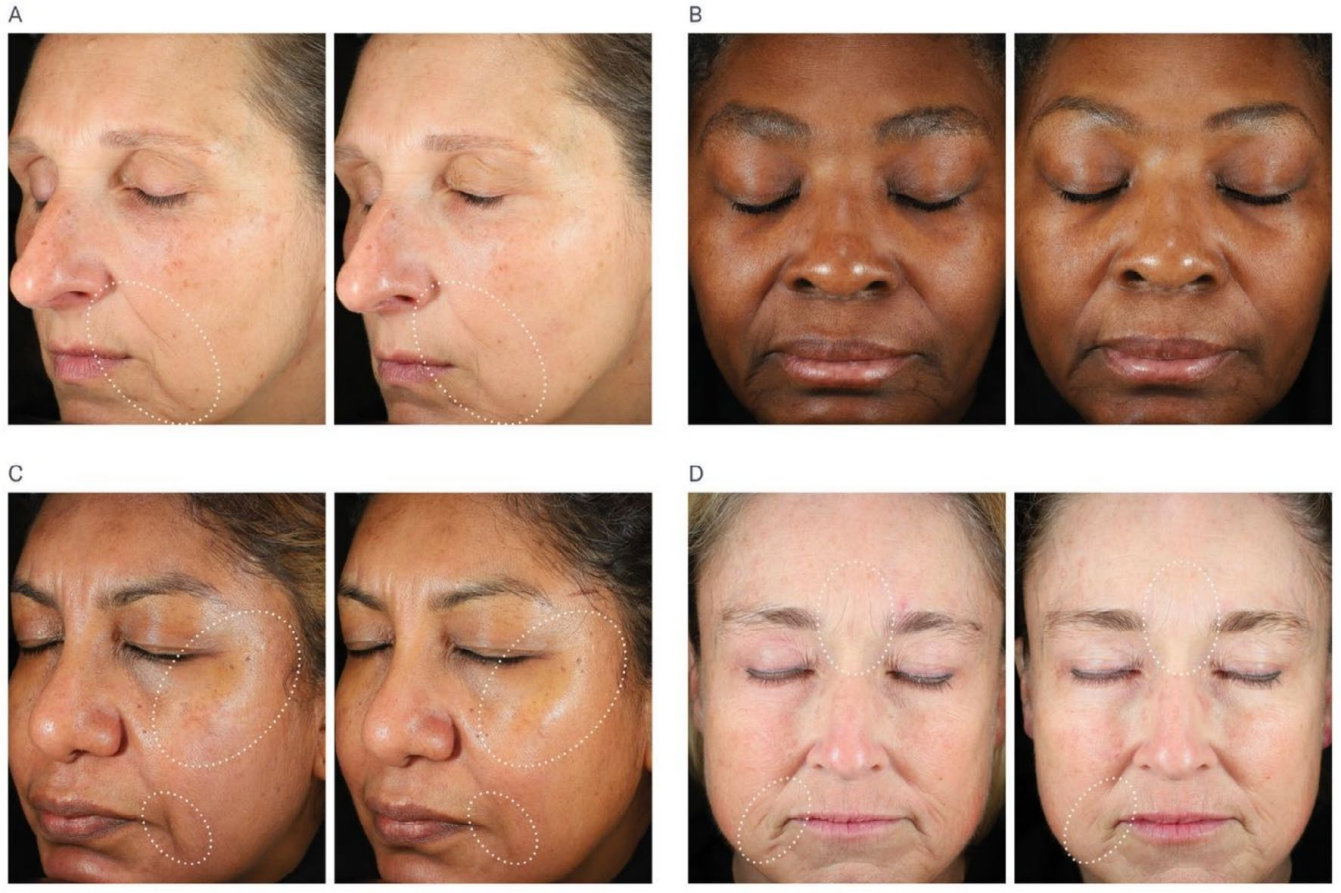
Unretouched clinical photography shown on clean, product‐free skin using standardized digital imaging. Visible improvements demonstrated from baseline following use of MYS‐REV serum at (A) Week 8, average result for lines and wrinkles/elastosis; (B) Week 8, average result for dullness and lines/wrinkles shown; (C) Week 12, above average result for elastosis and dullness shown; and (D) Week 16, above average result for elastosis and lines/wrinkles shown.

Measurements for skin hydration and transepidermal water loss (TEWL) were significant after the initial application of MYS‐REV (35% and −19%, respectively, all *p* < 0.0001; *n* = 44) with sustained improvement through 16 weeks (60%, −27%, all *p* < 0.0001; Figures 3, 4).

### 3.3 Safety and Tolerability

There were no incidents of edema or dryness/flaking on investigator assessment of tolerability. Subjects reported a decrease in burning and stinging/tingling, with no change in itching at Week 16. All Adverse Events were mild in severity, and no subject discontinued the study due to an AE.

### 3.4 Subject Satisfaction

Subjects reported high levels of satisfaction as early as 4 weeks. More than 85% of subjects reported their skin looks or feels more hydrated, and at least 70% reported their skin looks rejuvenated, healthier, and rebalanced. After 16 weeks of use, 96% of subjects reported that the overall quality of their skin was improved; 94% felt their skin looks and feels more hydrated and feels smoother; 92% reported their skin looks rejuvenated and feels revitalized and soothed; and 90% reported their skin looks plumper and revived and feels reenergized.

## 4 Discussion

Patients and clinicians generally focus on menstrual irregularities and vasomotor symptoms (VMS; “hot flashes”) associated with the hormonal fluctuations related to the menopausal transition. However, the impact of menopause‐related hormonal fluctuations on skin, while substantial, is rarely discussed or addressed. In addition to the challenges of efficiently responding to general internal and external stressors, skin must also be able to adapt to the effects of the diminishing levels of estrogen, and particularly 17β estradiol. Estrogen plays a key role in maintaining skin health through its supportive effects on collagen and elastin synthesis, hydration, and in maintaining overall skin vitality. Notably, diminishing levels of 17β estradiol cause key functional and structural skin changes [2], including a reduction of collagen, elastin, and fibroblast function [3], which lead to fine lines/wrinkles, sagging, and crepey skin, an altered ceramide production [4] and decreased hydration [5], leading to skin dryness, impaired barrier function, and decreased antioxidant capacity [3, 6]. Other clinical manifestations resulting from menopause‐related hormonal fluctuations include skin dullness/sallowness, skin redness, and rough skin texture.

It is desirable to rebalance skin's functional pathways and restore premenopausal skin health to offset the changes resulting from menopause‐related hormonal fluctuations (as well as other intrinsic and extrinsic factors). Prescription topical estrogens (predominantly containing estriol and not 17β estradiol) or selective estrogen receptor modulators (SERMs), can benefit some women who have menopause‐related skin symptoms [16]. However, these options may not be appropriate for women with a history of, or at risk for, hormone‐dependent cancers, and they lack sufficient efficacy and safety data for long‐term use for skin concerns. Nonhormonal topical strategies, including agents with barrier strengthening ingredients (ceramides, fatty acids, cholesterol), humectants (hyaluronic acid, glycerin), AHAs and BHAs, retinoids, antioxidants, and emollients can improve the appearance of erythema, skin dullness, rough texture, uneven pigmentation and enlarged pores, but do not target the body's ability to reduce oxidative stress or restore balance to pathways responsible for barrier function and structural integrity [17, 18, 19]. In contrast, plant adaptogens are natural components or extracts that target multiple pathways, support the body's resistance to negative stressors, and foster balance [7].

A novel serum (MYS) was developed using proprietary P.A.T.H. technology that combined nine plant adaptogens shown to support skin's natural ability to achieve balance and visibly improve skin quality, leading to significant improvements in erythema, dullness, texture, pore size, and uneven pigmentation [8]. The enhanced serum (MYS‐REV) used in this trial was formulated with 13 plant adaptogens specifically selected for their demonstrated ability to unite the skin pathways impacted by hormonal fluctuations, such as dryness, dullness, wrinkles, and loss of firmness. The P.A.T.H.[13] biotechnology was combined with TAP technologies (Allyl PQQ; pyrroloquinoline quinone allyl ester) to restore and rebalance dry‐to‐very dry perimenopausal and postmenopausal skin.

The benefits of MYS‐REV manifested in objective clinically apparent facial improvements in elasticity, lines/wrinkles, erythema, dullness, texture/roughness, pore size, and uneven pigmentation. More than half (53%) of the subjects were at least 5 years postmenopause and thus had a greater degree of estrogen depletion, which may have contributed to the apparent delay in statistically significant improvements in elastosis/crepey skin and lines and wrinkles, as their skin likely required a longer time to respond to a topical serum. Application of MYS‐REV also led to immediate objective improvements in skin hydration and TEWL that were sustained through 16 weeks. Importantly, subjects reported high levels of satisfaction as early as 4 weeks, and throughout the study period. At 16 weeks, 94% of subjects reported their skin looks and feels more hydrated, 92% reported their skin looks rejuvenated and feels revitalized, and 90% agreed their skin looks plumper and revived and feels reenergized. AEs were mild in severity, and no subject discontinued the study due to an AE.

In conjunction with this study, we also performed a study that examined the histological effect of MYS‐REV from skin biopsies obtained from both sun‐exposed and sun‐protected skin in a subset of perimenopausal and postmenopausal women who volunteered for this study. Biopsies obtained from the 10 volunteers demonstrated significant upregulation of elastin, Collagen III, and Claudin 1 in both sun‐exposed and nonexposed skin, with substantial upregulation of Collagen I in sun‐exposed and significant upregulation in nonexposed skin. There were also significant decreases in both exposed and nonexposed skin in MMP‐2 and in nonexposed skin in MMP‐9 at 16 weeks compared to baseline. These findings, reported elsewhere, demonstrated support of hydration, skin barrier support, and elasticity.

Future studies would include a larger, more diverse population including women diagnosed with low estrogen and progesterone levels, women currently taking hormonal replacement therapies, and those presenting with severe elastosis and lines/wrinkles along with a control group.

## 5 Conclusions

Twice‐daily use of an enhanced serum comprised of 13 plant‐based adaptogens using P.A.T.H.[13] and TAP technologies demonstrated significant improvements in elasticity and lines and wrinkles, along with significant and sustained improvements in hydration in skin affected by hormonal fluctuations associated with menopause, as assessed by investigators over 16 weeks. Use of a skincare regimen targeting the pathways affected by menopause‐related hormonal fluctuations led to visible improvements in skin of perimenopausal and postmenopausal women.

## Funding

This study was supported by skinbetter science, a dermatological beauty brand of L'Oreal USA Inc.

## Ethics Statement

This study received ethical approval from the Sterling IRB (Approval #12374) on October 14, 2024.

## Consent

All participants have provided consent to participate in the study and to have their photographs appear in any publication stemming from the findings of the study.

## Conflicts of Interest

Drs. Draelos and Moradi were investigators on this study; Amber Smathers is an employee of skinbetter science.

## Data Availability

The data that support the findings of this study are available on request from the corresponding author. The data are not publicly available due to privacy or ethical restrictions.

## References

[1] Society for Women's Health Research , “Menopause Disparities: Prevalence and Health Impact Across the United States,” (2024), https://swhr.org/wp‐content/uploads/2024/03/swhr_factsheet_menopause_rev_0222‐1.pdf.

[2] A. V. S. Faria and S. S. Andrade , “Decoding the Impact of Ageing and Environment Stressors on Skin Cell Communication,” Biogerontology 26, no. 1 (2024): 3. 39470857 10.1007/s10522-024-10145-3

[3] E. D. Lephart and F. Naftolin , “Menopause and the Skin: Old Favorites and New Innovations in Cosmeceuticals for Estrogen‐Deficient Skin,” Dermatology and Therapy 11, no. 1 (2021): 53–69. 33242128 10.1007/s13555-020-00468-7 PMC7859014

[4] A. C. Kendall , S. M. Pilkington , J. R. Wray , et al., “Menopause Induces Changes to the Stratum Corneum Ceramide Profile, Which Are Prevented by Hormone Replacement Therapy,” Scientific Reports 12, no. 1 (2022): 21715. 36522440 10.1038/s41598-022-26095-0 PMC9755298

[5] M. J. Thornton , “Estrogens and Aging Skin,” Dermato‐Endocrinology 5, no. 2 (2013): 264–270. 24194966 10.4161/derm.23872 PMC3772914

[6] E. D. Lephart and F. Naftolin , “Factors Influencing Skin Aging and the Important Role of Estrogens and Selective Estrogen Receptor Modulators (SERMs),” Clinical, Cosmetic and Investigational Dermatology 15 (2022): 1695–1709. 36017417 10.2147/CCID.S333663 PMC9397534

[7] X.‐X. Liu , C.‐Y. Chen , L. Li , et al., “Bibliometric Study of Adaptogens in Dermatology: Pharmacophylogeny, Phytochemistry, and Pharmacological Mechanisms,” Drug Design, Development and Therapy 17 (2023): 341–361. 36776447 10.2147/DDDT.S395256 PMC9912821

[8] Z. D. Draelos , P. E. Grimes , J. Watchmaker , and D. B. Nelson , “A Multicenter Trial Evaluating a Serum Comprised of Plant‐Based Adaptogens Targeting Skin Quality,” Journal of Clinical and Aesthetic Dermatology 17, no. 2 (2024): 15–19. PMC10911267 38444422

[9] Z. D. Draelos , D. H. McDaniel , S. Yoelin , S. Pot , O. Sotir , and D. B. Nelson , “Evaluation of a New, Advanced Antioxidant Containing Topical Allyl Pyrroloquinoline Quinone: Analysis of Antioxidant Properties and Visible Effects in Subjects With Facial Photodamage,” Journal of Clinical and Aesthetic Dermatology 16, no. 4 (2023): 53–59. PMC10110291 37077928

[10] E. Cadenas and K. J. Davies , “Mitochondrial Free Radical Generation, Oxidative Stress, and Aging,” Free Radical Biology & Medicine 29, no. 3–4 (2000): 222–230. 11035250 10.1016/s0891-5849(00)00317-8

[11] P. E. Grimes and D. B. Nelson , “Evaluation of an Advanced Antioxidant and Double‐Conjugated Retinoid/AHA Cream in Participants With FST IV‐V,” Journal of Cosmetic Dermatology 23, no. 4 (2024): 1291–1297. 38406974 10.1111/jocd.16246

[12] R. E. Fitzpatrick , M. P. Goldman , N. M. Satur , and W. D. Tope , “Pulsed Carbon Dioxide Laser Resurfacing of Photoaged Facial Skin,” Archives of Dermatology 132, no. 4 (1996): 395–402. 8629842

[13] P. G. Sator , J. B. Schmidt , M. O. Sator , J. C. Huber , and H. Hönigsmann , “The Influence of Hormone Replacement Therapy on Skin Ageing: A Pilot Study,” Maturitas 39, no. 1 (2001): 43–55. 11451620 10.1016/s0378-5122(00)00225-5

[14] T. J. Phillips , Z. Demircay , and M. Sahu , “Hormonal Effects on Skin Aging,” Clinics in Geriatric Medicine 17, no. 4 (2001): 661–672. 11535422 10.1016/s0749-0690(05)70092-6

[15] M. Brincat , S. Kabalan , J. W. Studd , C. F. Moniz , J. de Trafford , and J. Montgomery , “A Study of the Decrease of Skin Collagen Content, Skin Thickness, and Bone Mass in the Postmenopausal Woman,” Obstetrics & Gynecology 70, no. 6 (1987): 840–845. 3120067

[16] E. K. M. Šabović , T. Kocjan , and I. Zalaudek , “Treatment of Menopausal Skin – A Narrative Review of Existing Treatments, Controversies, and Future Perspectives,” Post Reproductive Health 30, no. 2 (2024): 85–94. 38379168 10.1177/20533691241233440

[17] D. M. Reilly and J. Lozano , “Skin Collagen Through the Lifestages: Importance for Skin Health and Beauty,” Plastic and Aesthetic Research 8 (2021): 2.

[18] A. G. Panossian , T. Efferth , A. N. Shikov , et al., “Evolution of the Adaptogenic Concept From Traditional Use to Medical Systems: Pharmacology of Stress‐ and Aging‐Related Diseases,” Medicinal Research Reviews 41 (2021): 630–703. 33103257 10.1002/med.21743 PMC7756641

[19] A. Zorina , V. Zorin , D. Kudlay , and P. Kopnin , “Molecular Mechanisms of Changes in Homeostasis of the Dermal Extracellular Matrix: Both Involutional and Mediated by Ultraviolet Radiation,” International Journal of Molecular Sciences 23 (2022): 6655. 35743097 10.3390/ijms23126655 PMC9223561

